# The Oncogenesis of Glial Cells in Diffuse Gliomas and Clinical Opportunities

**DOI:** 10.1007/s12264-022-00953-3

**Published:** 2022-10-13

**Authors:** Qiyuan Zhuang, Hui Yang, Ying Mao

**Affiliations:** 1 Department of Neurosurgery, Huashan Hospital, Fudan University, Shanghai, 200040 China; 2 National Center for Neurological Disorders, Huashan Hospital, Fudan University, Shanghai, 200040 China; 3 Shanghai Key Laboratory of Brain Function Restoration and Neural Regeneration, Huashan Hospital, Fudan University, Shanghai, 200040 China; 4 Institute for Translational Brain Research, Fudan University, Shanghai, 200032 China; 5 State Key Laboratory of Medical Neurobiology and MOE Frontiers Center for Brain Science, Institute for Translational Brain Research, Institutes of Brain Science, Fudan University, Shanghai, 200032 China; 6 Neurosurgical Institute of Fudan University, Shanghai, 200032 China

**Keywords:** Glioma origin, Stem/progenitor cell, Oncometabolite, Immune heterogeneity, Neuron-tumor interaction

## Abstract

Glioma is the most common and lethal intrinsic primary tumor of the brain. Its controversial origins may contribute to its heterogeneity, creating challenges and difficulties in the development of therapies. Among the components constituting tumors, glioma stem cells are highly plastic subpopulations that are thought to be the site of tumor initiation. Neural stem cells/progenitor cells and oligodendrocyte progenitor cells are possible lineage groups populating the bulk of the tumor, in which gene mutations related to cell-cycle or metabolic enzymes dramatically affect this transformation. Novel approaches have revealed the tumor-promoting properties of distinct tumor cell states, glial, neural, and immune cell populations in the tumor microenvironment. Communication between tumor cells and other normal cells manipulate tumor progression and influence sensitivity to therapy. Here, we discuss the heterogeneity and relevant functions of tumor cell state, microglia, monocyte-derived macrophages, and neurons in glioma, highlighting their bilateral effects on tumors. Finally, we describe potential therapeutic approaches and targets beyond standard treatments.

## Introduction

Gliomas, traditionally named due to their close resemblance to glial cells, are the most frequent intrinsic primary tumors of the brain [1–3]. Different from other oncological diseases that benefit from multimodal therapy, limited progress has been made in the management of gliomas [4, 5]. Therefore, ongoing efforts to understand their highly heterogeneous nature and complicated reciprocal microenvironmental communication have been undertaken [6, 7]. Among their forms, diffuse gliomas, which have an unfavorable prognosis and high morbidity in adult patients, have been historically diagnosed as one of three categories outlined in the 2016 WHO central nervous system (CNS) classification [8, 9]: oligodendroglioma, astrocytoma, or glioblastoma (GBM). These subtypes share several molecular features and functional characteristics with their normal counterparts. Recent profiling efforts have identified subclassifications of diffuse gliomas by integrating histopathological analysis and genetic events [10]. Importantly, isocitrate dehydrogenase (IDH) status and chromosome 1p/19q co-deletion [11], have been identified as predictive genetic landmarks of favorable outcomes and have had a profound impact on treatment strategies and the design of clinical trials [12]. In particular, robust biomarkers have also been described by Eckel-Passow *et al.*, who classify gliomas into five principal groups with prognostic significance. Of these, the triple-negative gliomas (no mutations in IDH and TERT plus a 1p/19q non-codeletion) were the most prevalent in a Chinese cohort [13, 14]. Mutations of *TP53* and H3.3-K27M in triple-negative gliomas implicated an unfavorable prognosis [14]. Notably, the fifth edition of the WHO CNS (2021 WHO CNS5) grouped gliomas according to these genetic changes to enable a complete diagnosis [15]. Other molecular signatures, such as cell-cycle regulatory elements (CDKN2A/B) and epidermal growth factor receptor (EGFR), have also contributed to the illustration of oncogenic pathways. Progress in genomics has validated diverse genetic alterations harbored in diffuse gliomas, rendering glioma cells distinct from one another. The expression patterns of genetic mutations suggest that astrocytomas and oligodendrogliomas originate from abnormal glial progenitors or stem cells. These findings have led to the hypothesis that the cellular heterogeneity of gliomas is affected by the glial developmental process, intercellular signaling, and microenvironment stress. This review discusses new advances in oncogenic glial lineage, and reciprocal interactions in gliomas (i.e. with neurons and microglia), offering new insights into the potential development of effective treatments.

## Glioma Origin: From Neurogenesis to Oncogenesis

### Glioma Stem Cells

Among the components constituting tumors, glioma stem cells (GSCs) are highly plastic subpopulations bearing stemness properties and are thought of as the site of tumor initiation. Similar to neural stem cells (NSCs), GSCs have the ability to self-renew, differentiate and resist DNA damage [16–18]. A series of biomarkers have been identified in GSC populations: CD133 (PROM1), SOX2 (a transcription factor widely expressed in potent stem cells), OCT-4 (a transcription factor that plays an essential role in stem cell pluripotency), and Nestin (an intermediate filament protein). Several studies have shown that the expression of these molecular markers is closely associated with pluripotency and stemness in gliomas. By intracranial grafting as few as 100 CD133+ cells, tumors have been effectively produced and resembled the phenotype of the original tumor type, whereas no transplanted tumor was observed after injection of 10^5^ CD133– cells [19]. Ablation of *Nestin+* stem-like cells was not able to halt tumor progression, indicating the involvement of other factors [20]. CD133+ Notch1+ GSCs have also been reported to be located at the frontier of invasive tissues, exhibiting white-matter-tract tropism. The positive-feedback loop involving Notch-SOX2 controls the invasive phenotype of GSCs along white matter tracts [21]. The stem-cell activity of CD133+ cells has also been found in medulloblastomas, pilocytic astrocytomas, and gangliogliomas. Higher tumor grade is correlated with an increased fraction of CD133+ cells in tumor cultures [20]. In addition, the non-GSC population is induced to a newly converted GSC-like state after treatment with chemotherapeutic agents (e.g., temozolomide), and has a more invasive phenotype with higher implantation efficacy [22]. These findings focus attention on the cellular state of GSCs in gliomas. Lin *et al.* described a single axis of gene signatures in proliferating GBM cells, ranging from proneural GSCs to mesenchymal GSCs. Lineage tracing *in silico* supports the idea that mGSCs, which correlate with poor predicted survival, are the progenitors of pGSCs in IDH wild-type GBM [23]. *Via* enriching GSCs from primary GBM specimens, Richards *et al.* found that GSCs exist in two cellular states from the perspective of transcriptional programs: developmental and injury-response programs [24]. The astrocyte maturation gradient in tumor cells has also been implicated in the transformation of GSCs, which comprise the bulk of the tumor. Thus, understanding the evolution and differentiation of GSCs is essential for developing effective targeting therapies and identifying the source of heterogeneity in gliomas.

### Neural Stem/Progenitor Cells

**Fig. 1:**
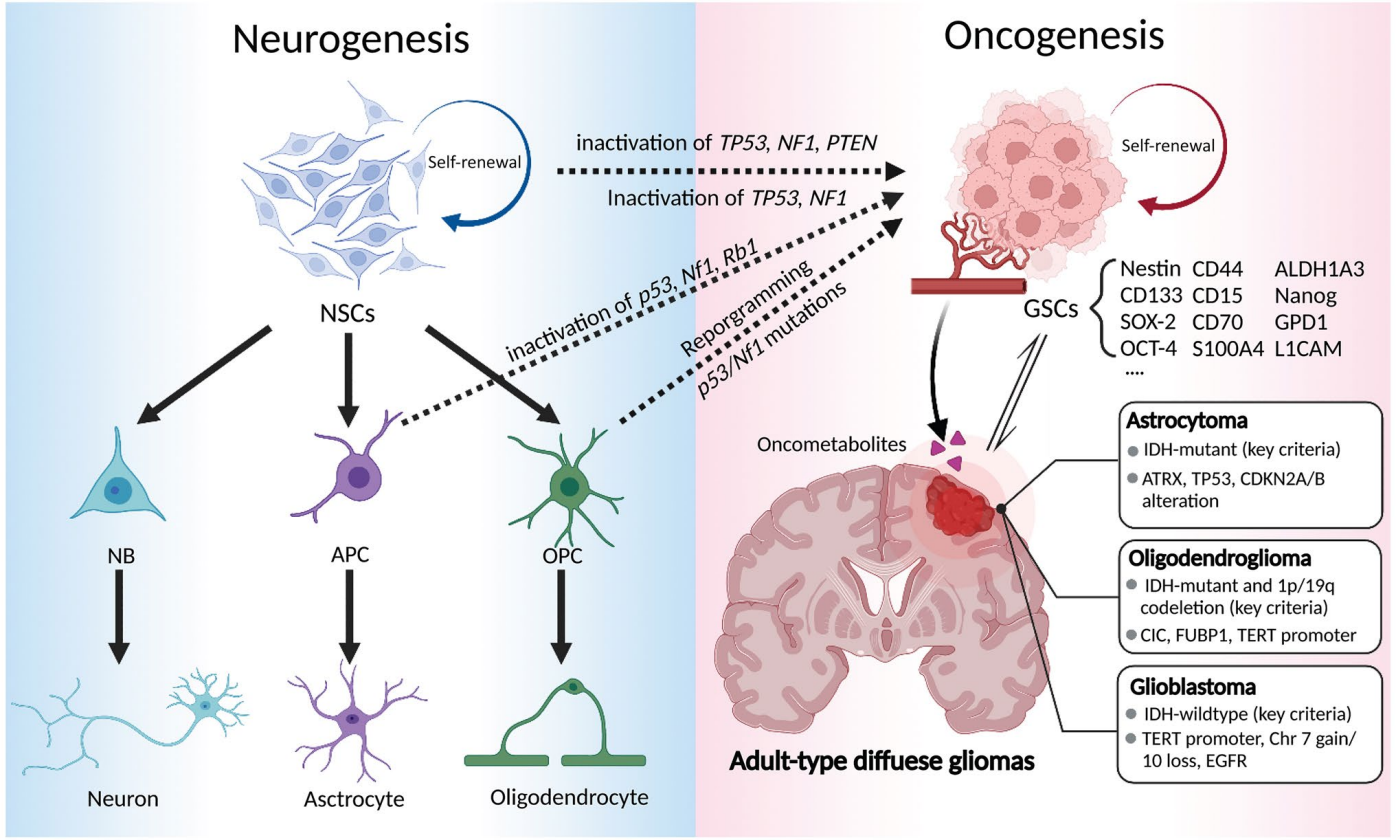
Glioma origin hypothesis. Left, schematic of the normal neurogenesis process in the brain. Neural stem cells differentiate into several types of progenitor cell, which can transform into neurons, astrocytes, and oligodendrocytes. Right, schematic of the potential oncogenesis process in the brain. Glioma-stem cells, which populate adult-type diffuse gliomas, are labeled with several reported biomarkers. Gliomas produce oncometabolites in the tumor microenvironment, which correspondingly stimulate their progression. The dashed line between neurogenesis and oncogenesis represents the reprogrammed molecular mechanisms that have been previously reported. NSCs, Neural stem cells; GSCs, Glioma stem cells, NBs, Neuroblasts; APCs, Astrocyte progenitor cells; OPCs, Oligodendrocyte progenitor cells.

Different from abnormal glioma stem cells that populate GBM, neural stem cells/progenitor cells (NSCs/NPCs) are the natural starting point for neuron/glial lineage development, and are highly regulated in the brain. It is essential to understand the tumorigenesis process and decipher the mechanisms through which glial developmental programs are used by tumor cells to populate the tumor. The largest NSC niches are located along the remote region of the lateral ventricles, named the subventricular zone (SVZ). These NSCs are relatively quiescent, maintain their stemness properties, and generate NPCs independent of the specific microenvironment around the perivascular niches. This complex microenvironment is composed of NPCs, oligodendrocyte progenitor cells (OPCs), astrocytes, microglia, macrophages, neurons, associated vasculature, and extracellular matrix. Interestingly, some typical markers of NSCs have been identified in GSCs such as Nestin, Sox2, CD44, and CD133 (Fig. 1) [25–27]. The striking similarities between NSCs and GSCs support the hypothesis that SVZ NPSCs play the role of apex cells in the hierarchy of gliomas. Chen *et al.* used a fluorescent reporter to label quiescent NSCs in the adult SVZ, and revealed the presence of neural stem-like cells in glioma tissue [28]. Deep genomic sequencing of a GBM patient cohort provided direct evidence for the hypothesis that astrocyte-like NSCs in the SVZ are the origin of GBM. More than 80% of patients diagnosed with GBM have tumor-free SVZ tissue that shares low-level driver mutations or cancer-driving genes with tumor samples [29]. Migration of astrocyte-like NSCs contributes to the generation of malignant gliomas in distinct regions of the brain [29]. Accordingly, genetically-engineered mouse models (GEMMs) are powerful tools for use in deciphering the lineage complexity of glial cells, and may reveal associations between progenitor cells and the broad spectrum of neoplasms throughout the brain. Parada *et al.* induced resultant malignant astrocytoma *via* early inactivation of *Tp53* and *Nf1* in mice [30], and demonstrated that manipulation of tumor suppressors (*Nf1*, *Tp53*, and *Pten*) in NSCs and NPCs *in vivo* is both necessary and sufficient for the formation of astrocytomas (Fig. 1). A recent study also showed that the histological and transcriptional heterogeneity of GBM is similar to genome-edited NSC-like cells such as sg*TP53/NF1/PTEN* or sg*TP53/NF1* in human pluripotent stem cells [31]. High-grade gliomas exhibit inactivation of *p16*^*INK4a*^/*p19*^*ARF*^ and activation of epidermal growth factor receptor (EGFR). Previous findings reported that co-deletion of *p16*^*INK4a*^/*p19*^*ARF*^ in NSCs with constitutive *EGFR* activation induce the phenotype of high-grade glioma [32]. However, no evidence of tumor formation was reported after targeting these GBM-relevant tumor suppressors in neuroblasts, late-stage neuronal progenitors, and differentiated neurons [33]. Jacques *et al.* reported that deletion of tumor suppressors genes (*TP53* and *PTEN*) in adult SVZ stem cells, but not astrocytes, gives rise to tumors. These studies imply that an increase in lineage restriction decreases the tumorigenic capacity of neuronal lineage cells [34]. Driving neuronal lineage differentiation is a potent antitumorigenic treatment strategy for GBM.

### Other Glial Lineage States

Combining single-cell sequencing (scRNA-seq) with advanced computational algorithms allows researchers to comprehensively analyze cellular states across tumors [35, 36]. Four cellular states, three of which are anchored in neurodevelopment, are found in diverse malignant cells of glioblastoma: OPC-like, NPC-like, astrocyte (AC)-like, and mesenchymal (MES)-like. These cellular states have the potential for tumor plasticity and are influenced by genetic drivers, providing an understanding of the heterogeneity and therapeutic resistance of GBM. It is well-known that tumor phenotype transitions are derived from stage-specific fate-switches and transcriptional alterations in progenitor cells. Newly-generated cells including ACs and oligodendrocytes, which are considered to be the two broad categories of CNS glia, are continuously produced in the subgranular zone and ventricular SVZ [37]. Both oligodendrocytes and ACs perform a variety of functions for maintaining CNS homeostasis.

Although the precursors of ACs have not been clearly defined, some ACs can reenter the cell-cycle following traumatic brain injury [38]. A recent study reported that ASCL1+EGFR+ apical multipotent intermediate progenitor cells generated by cortical radial glial cells in the SVZ and VZ can differentiate into glial cells and olfactory bulb (OB) interneurons. Those progenitor cells transform into AC-lineage restricted progenitor cells in late embryogenesis in mice [39]. Injection of ACs carrying oncogenes leads to the genesis of malignant gliomas. Furthermore, all tumors expressed markers expected in astrocytomas, such as Gfap [32, 40–42]. Combinations of deletions of *Pten*, *Tp53*, and *Rb1* in ACs in mature mice result in the progress of astrocytomas from grade III to grade IV [43]. Applying fluorescence-activated cell sorting–based strategy, a recent study reported five distinct AC subtypes across the adult brain and identified the specific subpopulations correlated with tumor invasion in gliomas [44]. OPCs, the most abundant cycling population in the adult CNS, is the last potential progenitor source of glioma origin. The OPC markers OLIG2 and NG2 are concurrently expressed in the major cycle-related cell population of the hippocampus [45]. Their correlates in mitotic characteristics give rise to the possibility that OPCs play a key role in tumorigenesis. Moreover, there is a large population of OLIG2+ cells (high Ki76 and CD133) in human gliomas, suggesting that proliferative OPCs may act as tumor-propagating cells [46, 47]. Several studies have found that oncogene mutations in OPCs are involved in the development of high-grade gliomas [48–50]. Overexpression of PDGF in OPCs, along with evidence of specific inactivation of *Nf1* and *tp53* in OPCs, are involved in the formation of malignant gliomas [51, 52]. Intriguingly, lineage tracing based on mosaic analysis with double markers (MADMs) has revealed that introducing *p53*/*Nf1* mutations only in OPCs, but not NSCs, consistently leads to oncogenesis (Fig. 1). Phenotypic and transcriptomic analyses have identified the salient OPC features of these tumors [53]. By applying lineage-targeted scRNA-seq, Weng *et al.* elegantly identified a primitive OPC intermediate population in the neonatal cortex. Reprogrammed OPCs transformed into a stem-like state, resulting in the development of malignant tumors [54]. These studies revealed that OPCs can directly generate GBM *via* stepwise genetic and epigenetic reprogramming. We summarize reported gene-edited mouse models that mimic different types of gliomas in Table 1.

**Table 1:** Gene-edited mouse models of gliomas.

	Driver genes	Methods	Original cells	Reference
Oligodendroglioma models	*v-erbB*/*Ink4a*/*Arf*^+/−^	Transgenic	*S100Aβ+*	Weiss *et al.* [153]
	*v-erbB*/*p53*^−/−^	Transgenic	*S100Aβ+*	Persson *et al.* [154]
	*H*-*Ras*/*EGFRvIII* (embryonic)	Transgenic	*GFAP+*	Ding *et al.* [155]
	PDGFB/*Ink4a-Arf*^−/−^	RCAS/*tv-a*	*Nestin*+	Dai *et al.* [156]; E Tchougounova *et al.* [157]
	PDGFB/*Akt*	RCAS/*tv-a*	*Nestin*+	Dai *et al.* [158]
	PDGFB	*In utero* intraventricular injections	Embronic neural precursors	Calzolari *et al.* [159]
	PDGFB	RCAS/*tv-a*	OPCs	Lindberg *et al.* [50]
	PDGFB-HA	RCAS/*tv-a*	*Nestin*+	Shih *et al.* [173]
	PDGF-A_L_	Transgenic	*GFAP-Cre+*	Nazarenko *et al.* ^[160]^
Astrocytoma models	*c-Myc*	Transgenic	*GFAP+*	Jensen *et al.* [161]
	*K-Ras*^*G12D*^	Transgenic	*GFAP+*	Abel *et al.* [162]
	*H*-*Ras*	Transgenic	*GFAP+*	Shannon *et al.* [163]
	*H*-*Ras*/*Pten*^fl/fl^	Transgenic	*GFAP-Cre+*	Wei *et al.* [164]
	*Pten*^*fl/fl*^*/Rb1*^*fl/fl*^*/Tp53*^*fl/fl*^	Transgenic	*GFAP-Cre+*	Chow *et al.* [43]
	*EGFR*/ *Ink4a-Arf*^−/−^	RCAS/*tv-a*	Nestin+/GFAP+	Holland *et al.* [165]
	*cisNf1*^*fl/+*^/*Trp53*^*+/−*^	Transgenic	*GFAP-Cre+*	Zhu *et al.* [30]
Glioblastoma models	PDGFB/Trp53^−/−^	Transgenic	*GFAP+*	Hede *et al.* [166]
	*K-Ras*/*Akt*	RCAS/*tv-a*	*Nestin*+	Holland *et al.* [167]
	*K*-*Ras*/ *Ink4a-Arf*^−/−^	RCAS/*tv-a*	*Nestin*+/ *GFAP*+	Uhrbom *et al.* [168]
	*K*-*Ras*/Akt/Pten^fl/fl^	RCAS/Cre	*Nestin-Cre*+	Hu *et al.* [169]
	*Cdkn2a*^*fl/fl*^*/Atrx*^*fl/fl*^*/Pten*^*fl/fl*^	RCAS/Cre	*PDGFRA/IDH*^*R132H*^*-Cre+*	Philip *et al.* [63]
	*Pten*^fl/+^/*cisNf1*^*fl/+*^/*Trp53*^+/−^	Transgenic	*GFAP-Cre+*	Kwon *et al.* [170]
	*P53*^*fl/fl*^/*Pten*^*fl/+*^	Transgenic	*GFAP-Cre+*	Zheng *et al.* [171]
	sg*TP53/NF1/PTEN* or sg*TP53/NF1*	CRISPER/Cas9 system	hNSCs	Wang *et al.* [31]
	sg*PTEN*/*NF1*, sg*TP53*/ *PDGFRA*^*Δ8–9*^	CRISPER/Cas9 system	iPSCs	Koga *et al.* [172]
	*Rb*^*fl/fl*^/*p53*^*fl/fl*^, *Rb*^*fl/fl*^/*p53*^*fl/fl*^/*Pten*^*fl/fl*^	Transgenic	*Adeno*-*Cre+*/*Adeno GFAP*-*Cre+*	Jacques *et al.* [34]
	Concurrent *p53*/*Nf1* mutations	Mosaic analysis with double markers	OPCs	Liu *et al.* [53]
	*p53*^−/*fl*^/*NF1*^*fl/fl*^	Transgenic	*NG2*-Cre+	Galvao *et al.* [52]

### Oncometabolites

Mutation at Arg^132^ of IDH1 was thought to be an early initiating event driving the evolution of gliomas [55, 56]. Mutation of IDH enzymes results in the elevation of (R)-2-HG levels from 1 mmol/L to 3 mmol/L at the center of IDH mutant gliomas [57]. 2-HG, known to be an important oncometabolite, is a competitive substrate of α-ketoglutarate-dependent epigenetic enzymes [58–60]. A high concentration of 2-HG *in vivo* inhibits histone lysine demethylases and TET hydroxylases, leading to impairment of DNA demethylation and eventual hypermethylation in gliomas [61]. Intriguingly, accumulation of (R)-2-HG also causes impairment of collagen protein maturation, which is associated with the endoplasmic reticulum stress response and basement membrane aberrations, leading to a microenvironment favorable to gliomas [62]. Notably, expression of *IDH1*^*R132H*^ cooperates with platelet-derived growth factor A expression and loss of *Cdkn2a*, *Atrx*, and *Pten* in glioma to mimic the proneural subtype of human GBM, which exhibits a stronger GBM formation ability *in vivo* [63]. By enhancing D-2-hydroxyglutarate-mediated DNA methylation, conditionally expressing *IDH1*^*R132H*^ in the NPCs of the murine SVZ increases the number of NSCs and their progeny. Regulated stem cells exhibit invasive characteristics and uncontrolled expansion, which may explain the process of oncogenesis in the early phase [64]. Platten’s research group conducted a phase I clinical trial in which 33 patients received treatment with an IDH1-specific peptide vaccine. The convincing clinical data showed that the vaccine is safe, and in terms of therapeutic effect, the IDH1-vaccine significantly prolongs the survival time of patients [65, 66]. These findings support the hypothesis that mutations in oncogenic metabolic enzymes dramatically affect the cellular status of gliomas, leading to mutations in other genes that collectively affect tumor transformation and promote tumorigenesis [67, 68]. Moreover, other groups have reported that tumor-derived kynurenine, IDO1, tryptophan 2,3-dioxygenase, and IL-4I1 mediate immunosuppressive activities in GBM [69–71]. Therefore, inhibitor therapy against these targets might be an alternative approach.

## Interactions Between Glioma and Microglia/Macrophages

### Heterogeneity Between Resident Microglia and Monocyte-Derived Macrophages (MDMs)

The microenvironment of gliomas consists of multiple interacting networks among cells, in which brain-resident microglia and infiltrating monocytes/MDMs contribute to a large fraction of the glioma immune landscape [72]. Microglia, derived from hematopoietic precursor cells of the yolk sac in the early developmental period, are crucial residential innate immune cells of the brain [73, 74]. They have an important influence in supporting neurogenesis, scavenging apoptotic cells, and refining synapses [73, 74]. Notably, different stages of glioma lead to differential compositions of the myeloid cell landscape. GBM can lead to partial disruption of the blood-brain barrier, enabling monocytes/MDMS to infiltrate the tumor. These distinct populations, termed tumor-associated macrophages (TAMs), have been widely reported as an important factor impinging on the intrinsic characteristics of tumor progression [75]. Using the head-protected irradiation and fluorescently tagged cell lineage tracing technique, microglia expressing high CD45 represent an inherent part of a glioma, while infiltrated tagged TAMs constitute up to 25% of the myeloid cell fraction after 21 days of tumor implantation [76]. The heterogeneity of time-lapse and spatial distribution in gliomas have been described through multiple timepoints and regional microdissection by scRNA-seq [77, 78]. Antunes *et al.* established the microglial fate-mapping system and revealed the similarities and differences in TAM distribution in newly-diagnosed GBM, recurrent GBM, and mouse GL261 models [79]. Moreover, microglia-derived TAMs or MDMs extracted from tumors are self-renewing populations that are unable to induce CD4^+^ T-cells or CD8^+^ T-cells, and compete for space in the tumor environment. Accordingly, the dominant myeloid population in glioma can progress from microglia-derived in the early phases to a mixture of microglia-derived TAMs and outnumbered MDMs in the later phase [89]. In addition, the aryl hydrocarbon receptor in monocytes boosts monocyte recruitment, and blocks antigen presentation expression in MDMs *via* the transcription factor KLF4 [69].

**Fig. 2:**
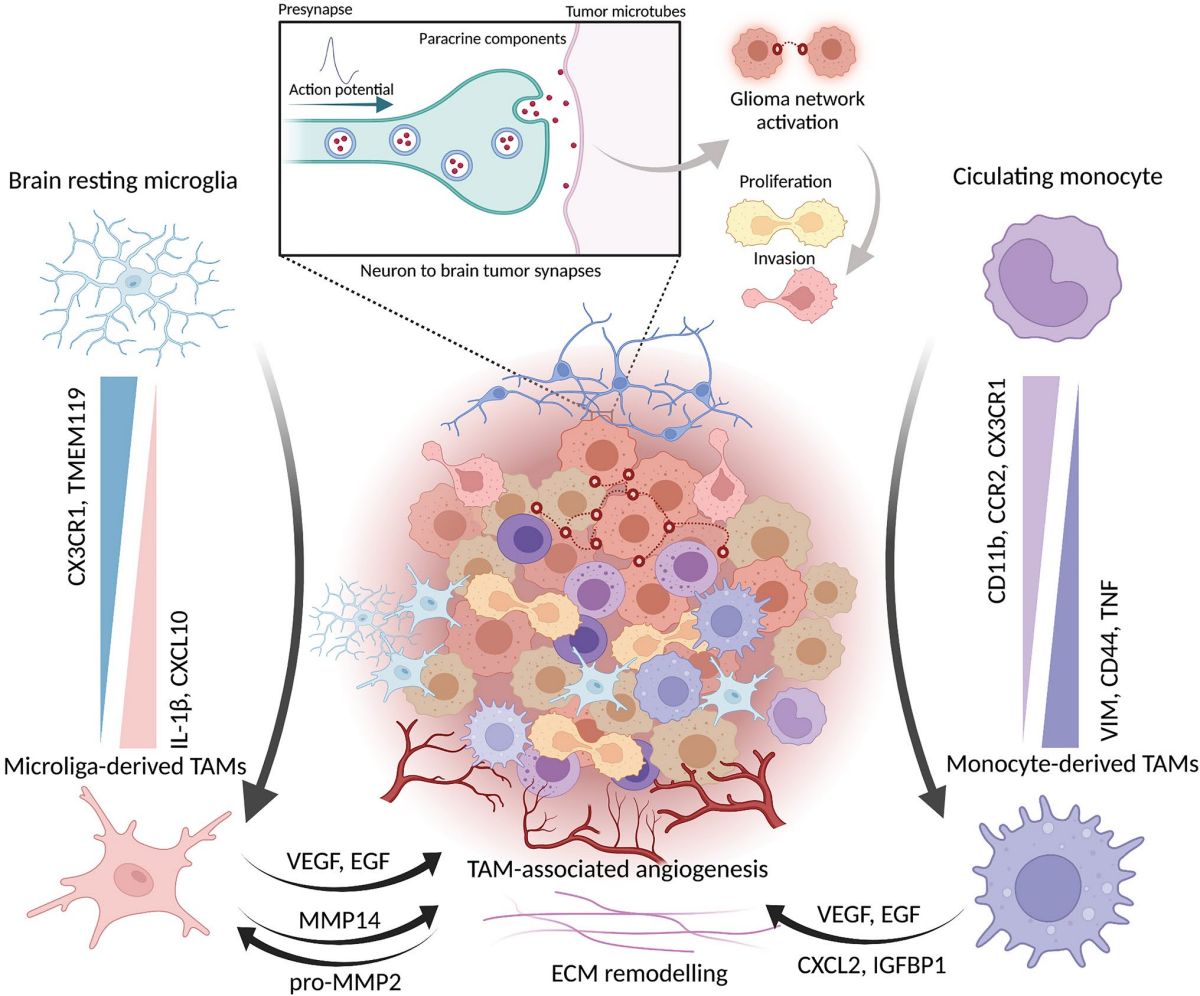
Reported communications in the glioma microenvironment. The tumor microenvironment of gliomas is complicated. Left, the relationship between brain resting microglia and microglia-derived TAMs. Wedges indicate differential biomarker expression between the two groups. The classical biomarkers of microglia (CX3CR1 and TMEM119) are reduced in microglia-derived TAMs, rather than other activated markers (IL-1β, CXCL10). Right, the relationship between circulating monocytes and monocyte-derived TAMs. Upper, schematic of neuron-to-brain tumor synapses. Lower, signaling pathways between TAMs and tumor cells. VEGF, vascular endothelial growth factor; EGF, epidermal growth factor; TAM, tumor-associated macrophage; ECM, extracellular matrix.

Considering the distinct biology of the two cell populations, it is essential to identify stable biomarkers to distinguish these two groups. In humans, microglia and macrophages can be classified *via* fluorescence-activated cell sorting using CD45 and CD49D (known as α4 integrin and ITGA4) [75]. Accurate separation in mice can be obtained by Ly6C, CD11b, F4/80, CD45, and Cx3cr1 [79]. As previously reported, the classical signature markers for microglia (P2ry12 and Sall1) and MDMs (Ly6c and Ccr2) are reduced during glioma-induced activation or differentiation and are insufficient for use in classifying the two populations [6]. After infiltration into the CNS, MDMs has a higher microglia signature gene pattern (Cx3cr1 and Tmem119) and lower CD45 (Fig. 2). Nevertheless, Qian *et al.* found that Crybb1 and Ldhb are specific and stable markers across different tumor stages in mice, and the cluster of MDMs consistently featured with Iqgap1 corresponded to other clinical datasets [80]. A variety of cytokines (IL-6 and IL-10) and several genes encoding chemokines associated with wound healing (Ccl22, Ccl17, Cxcl2, and Cxcl3) are upregulated in TAMs [81]. Of interest, a pro-inflammatory subset of microglia-derived TAMs was found to exhibit increased expression of Il1b (encoding IL-1β), Ifnb1 (encoding IFNβ1), Ccl4 (encoding C-C Motif Chemokine Ligand 4), Il12 (encoding IL-12), and Tnf (encoding TNF) [82, 83]. In addition, time-of-flight mass cytometry was also combined to reveal the heterogeneity of TAMs in gliomas [79]. Multiple subsets were identified exhibiting downregulation of classical microglial signature genes and, to the contrary, with upregulation of pro-inflammatory cytokines, responses to type I interferons, and hypoxia-associated molecules [79]. Collectively, these results describe novel glioma-associated microglia phenotypes and their diverging functions, which may provide new potential avenues for therapeutic interventions.

### Functional Characteristics of Microglia/Macrophages in Glioma

The role of microglia in glioma is controversial. In a model of organotypic slice cultures and *in vivo* implantation, the ablation of microglia impairs tumor growth and prolongs the survival of tumor-bearing mice. Further studies have revealed that glioma cells activate microglia but impair phagocytic activity [84–86]. Moreover, endogenous microglia derived from non-glioma subjects have a strong inhibitory effect on the expression of genes relevant to the cell cycle in tumor-initiating cells [87]. Microglia-activating substances such as GM-CSF and LPS can stimulate glioma cell migration cooperatively with endothelial cells, revealing that microglia do not merely react to tumor invasion but play a more complicated role in gliomas [88].

Compelling evidence underpins the perspective that genetic and molecular subtypes of GBM reflect distinct tumor microenvironment (TME), while secreted molecules or subsequently activated signaling from TAMs reciprocally remodels the cellular state of tumors. The functional interactions between GBM cells and components in the microenvironment play an important role in the modulation and infiltration of the brain. Liu *et al.* used scRNA-seq to characterize cell populations from IDH-WT and IDH-mutant samples and showed that the percentage of microglia and macrophages was higher in IDH-WT GBM [89]. Further, ~500 genes were found to be differently expressed in microglia isolated from IDH-WT and IDH-mutant samples [90]; but this cannot be exclusively considered as microglial heterogeneity. Using longitudinal scRNA-seq, Friedrich *et al.* examined myeloid cellular states in gliomas and demonstrated that differentiation of myeloid cells in IDH-mutant tumors is blocked by re-orchestration of tryptophan metabolism, leading to an immature phenotype [91]. Alteration of tryptophan metabolism in IDH-mutant gliomas reverses immunosuppression. It has also been reported that the mesenchymal subtype of GBM reported in The Cancer Genome Atlas is associated with an inferior prognosis and contains a higher proportion of TAMs compared to proneural or classical subtypes [36]. In addition, copy number amplifications such as *CDK4*, *EGFR*, and *PDGFRA* loci or mutation of the *NF1* locus are correlated with different cellular states in GBM [36]. Mutations of *NF1* result in a reduction of NF1 expression, which is predominantly found in the mesenchymal state of GBM, and are possibly responsible for the increase of TAM infiltration by NF1-regulated microglial chemotaxis [36]. Nevertheless, Hara *et al.* leveraged single-cell RNA sequencing and a mouse model to recapitulate mesenchymal GBM cellular states *in vivo*, exploring the crosstalk between macrophages and GBM subtype states. Analysis of ligand-receptor pairs suggests that oncostatin M in macrophages activates STAT3 signaling to induce a mesenchymal GBM cellular state *via* interaction with its receptors (or leukemia inhibitory factor receptor, in complex with GP130) in glioblastoma cells [92]. Moreover, in mouse GBM models, TAMs increase the levels of antigens presented, such as major histocompatibility complex type II expression, suggesting that TAMs in GBM can process antigens to T cells but are unable to activate the subsequent reaction [79]. Matrix metalloproteinases (MMPs) have been reported to be another group of proteins that are crucial to cells infiltration. MMP2 has been reported to be a marker of poor prognosis, facilitating the invasive and angiogenic properties of gliomas [93]. MMP2 is released in the form of pro-MMP2 and subsequently cleaved by MMP14 and converted into an active state which regulates the degradation of the extracellular matrix. In TME, the cleaved substrate pro-MMP2 is secreted by GBM cells, while microglia are the major source of MMP14 in TME. This reciprocal cooperation has been found to be regulated by its downstream signaling receptor TLR2 [94] and extracellular vesicles derived from GBM.

Furthermore, abundant and aberrant neovascularization is one of the defining characteristics of GBM. The process of angiogenesis and angiogenic factors have been extensively described. Resident microglia and peripheral macrophages collectively constitute perivascular niches, while a variety of pro-angiogenic molecules have been found to be upregulated. The CXCL2-CXCR2 signaling pathway is significantly upregulated during angiogenesis and has been shown to have a stronger angiogenic effect than VEGF *in vitro* [95]. Inhibiting the CXCL2-CXCR2 signaling pathway or selectively reducing resident microglia after tumor implantation decreases the tumoral vasculature count and tumor volume. Nevertheless, insulin-like growth factor-binding protein 1 (IGFBP1), released by microglia, is a novel factor mediating macrophage colony-stimulating factor-induced angiogenesis in GBM *via* an SYK-PI3K-NFκB-dependent mechanism [96]. Of interest, STAT3 expression (known as IL6/JAK/STAT3 signaling) in the glioma cell affects a variety of targeting genes and can propagate tumorigenesis by facilitating proliferation and angiogenesis. STAT3 upregulation, closely associated with abundant microglia and macrophages, is preferentially enriched in MES-like GBM. Further, ablation of receptors for advanced glycation end products (RAGE) prolongs the overall survival in a GL261 mouse model, and reduces the angiogenic factors secreted by TAMs. It has also been recently reported that microglial neuropilin-1 regulates vascular morphogenesis and affects its receptor VEGFR2 [97]. Administration of the inhibitor EG00229, which impairs the binding of tuftsin (Thr-Lys-Pro-Arg) with Nrp1, reverses the anti-inflammatory state of microglia through transforming growth factor beta signaling [97, 98]. Thus, the interactions between TAMs and GBM are complicated and multifactorial, and understanding the subtypes of TAMs presented in different primary gliomas is important for screening and developing subtype-specific targets.

## Neurobiology of Gliomas

Epileptic seizures, memory disorders, and cognitive impairment are common manifestations of patients with gliomas. These clinical characteristics have long been thought of as the result of mechanical pressure caused by the occupying lesion, while little is known about the interactions between tumor cells and surrounding neurons. However, a close relationship between tumors and the CNS has been validated in that gliomas exhibit electrical activity and are integrated into neural networks [99], which is thought to be a milestone event in the rapidly emerging field termed cancer neuroscience [100]. Compared to other pro-tumor factors derived from adjacent normal cells, the supportive influence of neurons includes direct (electrical, synapses, or synapse-like structures) and indirect (chemical) effects. Using electron microscopy, Venkataramani *et al.* described subtypes of distinct synapses formed by gliomas [101]. There are three morphological categories of neuron-glioma synapses that are consistently formed in incurable human gliomas but hardly exist in oligodendrogliomas. A parallel study found broad expression of glutamate receptor genes in high-grade gliomas, including IDH-mutant glioma, IDH-wild-type glioma, and diffuse intrinsic pontine glioma. Targeted patch-clamp recordings showed the existence of spontaneous excitatory postsynaptic currents that are mediated by glutamate receptors of the AMPA subtype. Synchronized Ca^2+^ transients are generated by neuronal firing, while genetic perturbations of AMPA receptors or the AMPA receptor antagonist perampanel reduce the invasiveness of gliomas [101]. These studies suggest direct, biologically relevant glutamatergic communication between neurons and glioma cells (Fig. 2). Excessive glutamate released by glioma cells may explain the recurrent seizures in patients. Intriguingly, it has been reported that the expression of glutamate transporters is increased in para-tumor cells, and performs a neuroprotective function in animal models [102]. In addition, excessive glutamate release may also lead to opening of the blood-brain barrier *via* the activation of N-methyl-D-aspartate receptors [103], and this is beneficial to the efficacy of drug delivery. In a recent impressive study, Chen *et al.* used an autochthonous mouse model to recapitulate adult OPC-originated gliomagenesis and found that olfaction can directly regulate gliomagenesis *via* insulin-like growth factor 1 (IGF1) signaling [104]. The activity of olfactory receptor neurons (ORNs) has significant effects on the progress of gliomas, while specific knockout IGF1 receptors in mutant OPCs abolishes the influence derived from ORNs. According to these groundbreaking studies, gliomas have the ability to form electrical and functional synapses with surrounding neurons, which drive tumor growth and resistance [105, 106].

Notably, tumor cells from incurable gliomas share several features with developing neurons (in the process of axonal and dendritic outgrowth) and extend long and thin microtubes [105]. Several reports have found that neurotransmitters in TME drive tumor growth and invasion. Nevertheless, Venkatesh and colleagues [107, 108] revealed a novel mechanism behind this reciprocal influence, showing that neuron paracrine secretion of neuroligin-3 (NLGN3) facilitates tumor progression and in turn induces a synaptic gene signature in the tumor cell. Researchers applied the optogenetic approach *in vivo* and *in vitro*, demonstrating that the firing activity of neurons promotes the proliferation and growth of glioma cells. Moreover, NLGN3, secreted by cortical projection neurons and oligodendrocyte precursor cells, is the leading candidate mitogen regulating this process. NLGN3 is broadly expressed in excitatory synapses and affects glioma proliferation through the phosphoinositide 3-kinase–mammalian target of rapamycin pathway [107]. Remarkably, the growth of GBM xenografts is significantly impaired in *Nlgn3*-knockout mice. In addition, brain-derived neurotrophic factor (BDNF) has also been validated to play a central role in classical synaptic functions and has a stimulating effect in TME [109].

Of interest, the interactions between glioma and neurons might involve the intimate interplay of neurons with precursor cells (NPCs and OPCs). Neuron-to-non-neuron synapses were first described by Bergles *et al.* in 2000. They reported that neurons form *bona fide* synapses with OPCs and regulate their proliferation [110]. Electrophysiological analyses revealed that these neuron-glial synapses are similar to normal neuron-neuron synapses, sharing features such as rapid activation, quantal responses, facilitation, depression, and presynaptic inhibition. Previous evidence showed that gliomas mainly originate from NPCs and/or OPCs, which may explain these structural similarities. Moreover, Elizabeth *et al.* reported that NPCs in the SVZ stimulate invasion of glioma cells through the secretion of chemoattractant signals. Inhibition of Rho/ROCK signaling reduces invasion of glioma cells induced by factors secreted by SVZ NPCs [111]. This novel framework provides new insights into understanding the progression of cancer and sheds light on therapeutic opportunities that can disrupt these communications.

## New Insights for Therapeutic Opportunities in High-Grade Glioma

### Immune Checkpoint Therapy

The treatment of high-grade glioma is still mainly based on surgery with postoperative radiotherapy and chemotherapy [112]. It is promising that some novel treatment strategies have shown high promise. Tumor immunotherapy has attracted much attention, but owing to the lack of specificity of brain immunity, current immunotherapy strategies require further improvement before application in high-grade gliomas [113, 114]. A series of clinical trials that tested the safety and efficacy of targeting immune checkpoints showed no improved survival benefit in GBM patients [115–117]. In 2017, a phase III clinical trial comparing nivolumab (PD1 monoclonal antibody) with bevacizumab (VEGFA monoclonal antibody) showed that patients with recurrent glioblastoma did not benefit from nivolumab treatment (CheckMate-143) [117]. A phase III clinical trial comparing nivolumab plus radiotherapy with standard chemoradiotherapy further confirmed that patients with *de novo* O-6-methylguanine-DNA methyltransferase (MGMT) unmethylated glioblastoma did not benefit from nivolumab therapy (CheckMate-498) [118]. In addition, CheckMate-548 yielded similar negative results in a phase III trial which compared nivolumab plus standard chemoradiation *versus* standard chemoradiation in patients with MGMT-methylated glioblastoma [119]. Alternatively, a recent study demonstrated that changing the dosing strategy and administering PD1 antibodies using neoadjuvant therapy can prolong the median survival of patients with relapsed glioblastoma [120]. However, immune checkpoint inhibitors are unable to reverse immune exhaustion in GBM [121]. Mass cytometry time-of-flight analysis revealed that macrophages contributed to 72.6% of the leukocytes in the TME [122], most of which expressed multiple immunosuppressive markers. These data indicate that immune suppressive macrophages are an important confounder for attenuation of the T-cell response. Further understanding of the immune microenvironment within brain tumors is needed to improve the clinical efficacy of immune checkpoint therapy.

### Cell-Based and Oncolytic Virus Therapy

Cell therapy based on chimeric antigen receptors (CARs), which involves grafting a specific designed receptor onto an effector cell, is also a research frontier in the treatment of high-grade gliomas [123]. Clinical trials targeting three antigens, EGFRvIII, HER2, and IL-13R alpha2, have confirmed that the application of CAR-T is safe, feasible, and potentially effective [124–127]. However, the application of CARs to brain tumors still faces challenges due to tumor heterogeneity and antigen loss. Antigen loss in recurrent tumors has been reported in both CAR-T therapy targeting EGFRvIII and IL-13R alpha2 [125, 127]. Interestingly, the major toxicity of CAR-T cells is cytokine release syndrome (CRS). Myeloid-derived macrophages have been found to contribute to the pathogenesis of CRS, mainly mediating the production of core cytokines including IL-6, IL-1, and interferon-γ [128]. In addition, Rodriguez-Garcia *et al.* demonstrated that CAR-T cell-mediated selective elimination of folate receptor β TAMs resulted in an increase in endogenous activated CD8+ T cells, decreased tumor burden, and prolonged survival [129]. Several reports have highlighted that engineering CAR macrophages is a valuable strategy in GBM. CAR-macrophages have been adapted and designed to produce pro-inflammatory cytokines, which convert subtypes of macrophages from M2 to M1. The polarization of macrophages increases T cell anti-tumor activity and further modulates the pro-inflammatory characteristics of TME [130, 131]. Thus, it is essential to develop new techniques to screen out suitable antigen sites of tumors or immunosuppressive cells, reduce antigen loss, and retard immune cell exhaustion.

Notably, therapeutic vaccination for brain tumors may be a promising treatment strategy. The EGFRvIII-based vaccine was successful in phase II clinical trials for glioblastoma, but failed to achieve positive results in phase III clinical trials. Tumor samples from relapsed patients showed immune escape, which is also a pressing problem during the vaccination treatment period and similar to the challenges of cell therapy [132–135]. Developing individualized vaccines based on specialized patient gene mutation patterns and expression profiles that collectively target the multiple glioma antigens is a potential future direction. Moreover, oncolytic virus therapy can activate antitumor immune responses, which are an important active immune therapy. A clinical trial using recombinant poliovirus in the treatment of recurrent glioblastoma suggested that this technique is effective and safe [136]. Therefore, improving the targeting of oncolytic viruses, slowing the clearance of oncolytic viruses by the immune system, and reducing the side-effects of oncolytic viruses are important methods for improving the clinical application value of oncolytic viruses.

### Transdifferentiation Induction and Glioma Reprogramming

Owing to the similarity between GSCs and NSCs researchers have proposed that inducing GSCs to differentiate into terminally-differentiated cells, especially neurons, might be a supportive strategy to inhibit the progression of brain tumors. It has been reported that inhibition of the Notch pathway can significantly induce a subset of patient-derived GSCs with high *ASCL1* expression to differentiate into neuron-like cells [137]. In addition, previous studies have shown that the pression of glioma cells can be retarded by inducing glial differentiation *via* activation of microRNA or BMP signaling [138, 139]. Overexpression of three neurogenic transcription factors (*ASCL1*, *BRN2*, and *NGN2*) reprogrammed 20%–40% of human glioma cells into TUBB3-positive neurons *in vitro* [140]. Cooperating with *NGN2* and *SOX11*, intravenous injection of overexpressing viruses has been shown to improve the reprogramming efficiency of human glioma cells into terminally-differentiated neuron-like cells, thus delaying tumor progression and significantly prolonging the survival of tumor-bearing mice [141]. A similar result was found *via* overexpression of *NGN2*, *ASCL1*, and *NeuroD1* in glioma cells. Unfortunately, gene regulation as a treatment for GBM are bound to face great challenges, and certain risks exist in the clinical application of transgenic technology and virus transfection such as off-target effects and neurotoxicity. However, these results suggest the potential for reprogramming of GBM cells into neurons [142–144].

### Disruption of Neuron-Glioma Communication

Glutamatergic synaptic structures and gap junctions have been identified in diffuse gliomas and can evoke long-lasting depolarizing currents, Ca^2+^ flux, and subsequent electrical network reactions. This cascade of electrical responses in glioma subpopulations ultimately promotes cell invasion and mitosis [99, 101]. Noninvasive brain stimulation (NiBS) is a group of techniques applied to the scalp that are broadly used in clinical practice to modulate neural activity *via* transcranial electrical or magnetic fields (transcranial magnetic stimulation, TMS; tumor-treating fields, TTFields). Interestingly, NiBS can increase or decrease neural activity depending on different stimulation patterns, of which the mechanisms are considered to involve the regulation of synaptic plasticity [145]. Long-lasting effects across multiple regions of the brain have been reported after stimulation with magnetic or electrical fields [145, 146]. NiBS has also been reported to induce effects such as the modulation of glutamatergic transmission, BDNF-dependent plasticity, and the regulation of pathway activity [147, 148]. Thus, disruption of communication between glioma and neurons is a promising area of study. Considering the efficacy and safety of NiBS, both TMS and TTFields have been approved for the treatment of several psychiatric diseases [145]. As for neoplasms, the landmark EF-14 trial, showed that TTFields plus maintenance Temozolomide (TMZ) resulted in an increase in overall survival in patients with newly diagnosed GBM compared to TMZ alone (20.9 *vs* 16.0 months with TMZ alone). Furthermore, no systemic adverse events are associated with the addition of TTFields (48% *vs* 44% with TMZ alone) [149, 150]. Indeed, TTFields deliver a low intensity (1–3 V/cm) at medium frequency (100–300 kHz) to the tumor region, alternating extra physiological currents which do not affect neural activity but rather impede cancer cell mitosis. The formation of microtubules, which are essential structures for neuron-glioma communications [101, 151], is disrupted by TTFields. Recent reports also revealed that TTFields induce an increased release of micronuclei from tumor cells, leading to activation of cGAS/STING and AIM2/Caspase-1 [152]. After treatment with TTFields, T-cell activation and clonal expansion have been reported in samples and derived from the upregulation of adaptive immunity. Taylor *et al.* genetically or pharmacologically blocked BDNF-TrkB signaling in a xenograft model of pediatric glioblastoma, abrogating the tumor-promoting effects of BDNF on synapses and prolonging survival [109]. Thus, growing evidence suggests that the application of NiBS may be used to suppress glioma progression and tumor-promoting neuronal communication.

## Discussion

Understanding of biology and immunology in gliomas has advanced at an impressive pace in recent years. The brain TME comprises heterogeneous populations of cells exhibiting differences in genetic characteristics and performing various modes of reciprocal interaction to mediate tumor initiation, progression, and therapeutic response. Combining advancing technologies in genetic engineering and sequencing enables promising capabilities in diagnosis and personalized treatment, and deciphering the origins of tumor-supporting cells at the single cell level as well. Studying developmental programs is a promising strategy for understanding the process of oncogenesis, and essential targets for disrupting disease progression or remission may be found. We here reviewed advances in gene-edited mouse models that mirror human disease and discussed potential glioma-initiated progenitor cells that may be used for further investigations. In addition, interactions between tumor subtypes, microglia, MDMs, and neurons in the brain TME play an important role in tumor progression. In light of many mechanisms of tumor/non-tumor cell crosstalk and their accompanying outgrowths, these implications in complex TME caused by these interactions are important components of the major driver in glioma biology. We here highlight a nascent but fast-growing field termed cancer neuroscience, which mainly focuses on the tumor-neuron network and its role in the progress of cancer growth. Interesting questions that remain to be answered include: (1) how to specifically target tumor cells in the tumor-neuron axis and integrate neural regulation methods into existing clinical strategy, (2) why and how tumors communicate with neurons in the brain, and (3) whether histopathological subtypes of glioma have a neuron communication preference. Lessons learned from TME suggest that disruption of the tumor/non-tumor cell dialogue could be helpful in inventing potential novel therapeutic approaches beyond standard treatment such as immune checkpoint inhibitors, cell-based biotechniques, and noninvasive brain stimulation. These therapies could potentially become the keystones of clinical practice in the future.
